# (*E*)-4-[(2-Hydroxy­benzyl­idene)amino]benzene­sulfonic acid

**DOI:** 10.1107/S1600536808015407

**Published:** 2008-05-30

**Authors:** Xinli Zhang, Zongxiao Li

**Affiliations:** a Department of Chemistry, Baoji University of Arts and Science, Baoji, Shaanxi 721007, People’s Republic of China

## Abstract

The title mol­ecule, C_13_H_11_NO_4_S, displays a *trans* configuration with respect to the imine C=N double bond. The central benzene ring directly linked to N and the hydroxyl group are disordered over two orientations [occupancies of 0.510 (16)/0.490 (16) and 0.528 (8)/0.472 (8), respectively]. The dihedral angle between the two aromatic rings is 23.3 (5)° for the major component and 18.3 (5)° for the minor component. There is an intra­molecular O—H⋯N hydrogen bond and mol­ecules are linked into chains along the *a* axis by O—H⋯O hydrogen bonds.

## Related literature

For bond-length data, see: Allen *et al.* (1987 ▶).
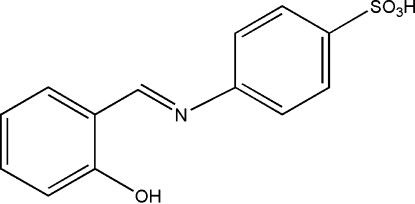


## Experimental

### 

#### Crystal data

C_13_H_11_NO_4_S
*M* *_r_* = 277.29
Monoclinic, 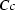

*a* = 4.8711 (5) Å
*b* = 29.022 (3) Å
*c* = 9.0356 (17) Å
β = 97.223 (2)°
*V* = 1267.2 (3) Å^3^
*Z* = 4
Mo *K*α radiation
μ = 0.27 mm^−1^
*T* = 298 (2) K
0.42 × 0.31 × 0.15 mm

#### Data collection

Siemens SMART CCD area-detector diffractometer
Absorption correction: multi-scan (*SADABS*; Sheldrick, 1996 ▶) *T*_min_ = 0.897, *T*_max_ = 0.961
3185 measured reflections
1952 independent reflections
1656 reflections with *I* > 2σ(*I*)
*R*_int_ = 0.026

#### Refinement

*R*[*F* ^2^ > 2σ(*F* ^2^)] = 0.050
*wR*(*F* ^2^) = 0.121
*S* = 1.09
1952 reflections
213 parameters
2 restraints
H-atom parameters constrained
Δρ_max_ = 0.34 e Å^−3^
Δρ_min_ = −0.30 e Å^−3^
Absolute structure: Flack (1983 ▶), 822 Friedel pairs
Flack parameter: −0.06 (14)

### 

Data collection: *SMART* (Siemens, 1996 ▶); cell refinement: *SAINT* (Siemens, 1996 ▶); data reduction: *SAINT*; program(s) used to solve structure: *SHELXS97* (Sheldrick, 2008 ▶); program(s) used to refine structure: *SHELXL97* (Sheldrick, 2008 ▶); molecular graphics: *SHELXTL* (Sheldrick, 2008 ▶); software used to prepare material for publication: *SHELXTL*.

## Supplementary Material

Crystal structure: contains datablocks I, global. DOI: 10.1107/S1600536808015407/ci2596sup1.cif

Structure factors: contains datablocks I. DOI: 10.1107/S1600536808015407/ci2596Isup2.hkl

Additional supplementary materials:  crystallographic information; 3D view; checkCIF report

## Figures and Tables

**Table 1:** Hydrogen-bond geometry (Å, °)

*D*—H⋯*A*	*D*—H	H⋯*A*	*D*⋯*A*	*D*—H⋯*A*
O2—H2⋯O3^i^	0.82	2.17	2.917 (5)	151
O4—H4⋯N1	0.82	2.01	2.665 (10)	136

Symmetry code: (i) 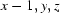.

**Fig. 1.**
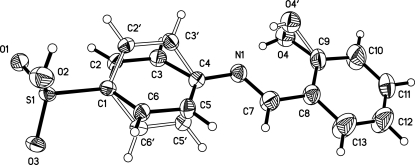
The molecular structure of the title compound. Displacement ellipsoids are drawn at the 30% probability level. Both disorder components are shown.

## supplementary crystallographic information

### Comment

Schiff base compounds have been of great interest for many years. These compounds play an important role in the development of coordination chemistry related to catalysis and enzymatic reactions, magnetism and molecular architectures. As an extension of the work on the structural characterization of Schiff base compounds, the crystal structure of the title compound is reported here.

The structure of the title molecule is shown in Fig. 1. The bond lengths and angles are within normal ranges (Allen *et al.*, 1987). The C7?N1 bond length of 1.262 (7) Å conforms to the value for a double bond. The dihedral angle between C1—C6 and C8—C13 benzene rings is 23.3 (5)° and that between C1/C2'/C3'/C4/C5'/C6' and C8—C13 rings is 18.3 (5)°. The C7—N1—C4—C5, C7—N1—C4—C3, O1—S1—C1—C6, O3—S1—C1—C6 and O2—S1—C1—C6 torsion angles are 22.8 (12)°, -156.4 (9)°, 173.5 (9)°, -58.9 (9)° and 56.3 (9)°, respectively. The molecule adopts a *trans* configuration about the C7?N1 bond. There exists an intramolecular O4—H4···N1 hydrogen bond involving the hydroxyl group and the imine N atom (Table 1).

In the crystal structure, the molecules are linked into chains running along the *a* axis by O—H···O hydrogen bonds.

### Experimental

Salicylaldehyde (0.1 mmol, 12.2 mg) and sulfamide (0.1 mmol, 17.2 mg) were dissolved in methanol (10 ml). The mixture was stirred at room temperature for 10 min and then filtered. The filtrate was allowed to stand in air for 3 d, after which time yellow block-shaped crystals of the title compound were formed by slow evaporation of the solvent. The crystals were collected, washed with methanol and dried in a vacuum desiccator using anhydrous CaCl_2_ (yield 54%). Analysis found: C 48.88, H 3.20, N 4.07%; calculated for C_13_H_11_NO_4_S: C 48.9, H 3.20, N 4.08%.

### Refinement

The central benzene ring is disordered over two orientations (C1—C6/C1,C2',C3',C4,C5',C6') with refined occupancies of 0.510 (16) and 0.490 (16). The –OH group is also disordered over two positions with refined occupancies of 0.528 (8) and 0.472 (8). H atoms were placed in geometrically idealized positions and constrained to ride on their parent atoms, with O—H = 0.82 Å, C—H = 0.93 Å and *U*_iso_(H) = 1.2*U*_eq_(C) and 1.5*U*_eq_(O).

### Crystal data

**Table:**

C_13_H_11_NO_4_S	*F*_000_ = 576
*M**_r_* = 277.29	*D*_x_ = 1.453 Mg m^−^^3^
Monoclinic, *C**c*	Mo *K*α radiation λ = 0.71073 Å
Hall symbol: C -2yc	Cell parameters from 1329 reflections
*a* = 4.8711 (5) Å	θ = 2.7–23.9º
*b* = 29.022 (3) Å	µ = 0.27 mm^−^^1^
*c* = 9.0356 (17) Å	*T* = 298 (2) K
β = 97.223 (2)º	Block, yellow
*V* = 1267.2 (3) Å^3^	0.42 × 0.31 × 0.15 mm
*Z* = 4	

### Data collection

**Table:**

Siemens SMART CCD area-detector diffractometer	1952 independent reflections
Radiation source: fine-focus sealed tube	1656 reflections with *I* > 2σ(*I*)
Monochromator: graphite	*R*_int_ = 0.026
*T* = 298(2) K	θ_max_ = 25.0º
φ and ω scans	θ_min_ = 1.4º
Absorption correction: multi-scan(SADABS; Sheldrick, 1996)	*h* = −5→5
*T*_min_ = 0.897, *T*_max_ = 0.961	*k* = −34→31
3185 measured reflections	*l* = −9→10

### Refinement

**Table:**

Refinement on *F*^2^	Hydrogen site location: inferred from neighbouring sites
Least-squares matrix: full	H-atom parameters constrained
*R*[*F*^2^ > 2σ(*F*^2^)] = 0.050	*w* = 1/[σ^2^(*F*_o_^2^) + (0.0495*P*)^2^ + 1.7913*P*] where *P* = (*F*_o_^2^ + 2*F*_c_^2^)/3
*wR*(*F*^2^) = 0.121	(Δ/σ)_max_ = 0.001
*S* = 1.09	Δρ_max_ = 0.34 e Å^−^^3^
1952 reflections	Δρ_min_ = −0.30 e Å^−^^3^
213 parameters	Extinction correction: none
2 restraints	Absolute structure: Flack (1983), 822 Friedel pairs
Primary atom site location: structure-invariant direct methods	Flack parameter: −0.06 (14)
Secondary atom site location: difference Fourier map	

### Special details

**Table:**

Geometry. All e.s.d.'s (except the e.s.d. in the dihedral angle between two l.s. planes) are estimated using the full covariance matrix. The cell e.s.d.'s are taken into account individually in the estimation of e.s.d.'s in distances, angles and torsion angles; correlations between e.s.d.'s in cell parameters are only used when they are defined by crystal symmetry. An approximate (isotropic) treatment of cell e.s.d.'s is used for estimating e.s.d.'s involving l.s. planes.
Refinement. Refinement of *F*^2^ against ALL reflections. The weighted *R*-factor *wR* and goodness of fit *S* are based on *F*^2^, conventional *R*-factors *R* are based on *F*, with *F* set to zero for negative *F*^2^. The threshold expression of *F*^2^ > σ(*F*^2^) is used only for calculating *R*-factors(gt) *etc*. and is not relevant to the choice of reflections for refinement. *R*-factors based on *F*^2^ are statistically about twice as large as those based on *F*, and *R*- factors based on ALL data will be even larger.

### Fractional atomic coordinates and isotropic or equivalent isotropic displacement parameters (Å^2^)

**Table:**

	*x*	*y*	*z*	*U*_iso_*/*U*_eq_	Occ. (<1)
S1	0.75870 (18)	0.76105 (3)	0.47671 (13)	0.0372 (3)	
N1	0.6129 (9)	0.96320 (13)	0.4070 (4)	0.0490 (10)	
O1	0.6035 (6)	0.73963 (10)	0.3515 (3)	0.0470 (8)	
O2	0.6306 (8)	0.74475 (13)	0.6247 (5)	0.0663 (11)	
H2	0.4678	0.7526	0.6185	0.099*	
O3	1.0490 (7)	0.75204 (11)	0.5021 (4)	0.0501 (8)	
O4	0.387 (2)	1.0337 (3)	0.2463 (11)	0.0675 (18)	0.528 (8)
H4	0.4579	1.0080	0.2483	0.101*	0.528 (8)
O4'	0.285 (2)	1.0279 (4)	0.3133 (12)	0.0675 (18)	0.472 (8)
H4'	0.3138	1.0031	0.3557	0.101*	0.472 (8)
C1	0.7196 (9)	0.82135 (14)	0.4573 (5)	0.0365 (11)	
C2	0.585 (3)	0.8377 (3)	0.3264 (16)	0.042 (3)	0.510 (16)
H2A	0.5178	0.8179	0.2493	0.051*	0.510 (16)
C3	0.554 (3)	0.8837 (3)	0.3133 (16)	0.045 (3)	0.510 (16)
H3	0.4598	0.8955	0.2254	0.055*	0.510 (16)
C2'	0.470 (3)	0.8413 (3)	0.3925 (16)	0.042 (3)	0.490 (16)
H2'	0.3224	0.8223	0.3586	0.051*	0.490 (16)
C3'	0.439 (3)	0.8887 (3)	0.3777 (16)	0.044 (3)	0.490 (16)
H3'	0.2733	0.9015	0.3339	0.053*	0.490 (16)
C4	0.6589 (11)	0.91570 (17)	0.4298 (6)	0.0426 (11)	
C5	0.805 (3)	0.8949 (4)	0.5579 (16)	0.046 (3)	0.510 (16)
H5	0.8868	0.9136	0.6346	0.056*	0.510 (16)
C6	0.829 (3)	0.8473 (4)	0.5726 (16)	0.043 (3)	0.510 (16)
H6	0.9179	0.8339	0.6592	0.052*	0.510 (16)
C5'	0.909 (3)	0.8993 (4)	0.4909 (19)	0.047 (3)	0.490 (16)
H5'	1.0547	0.9193	0.5213	0.056*	0.490 (16)
C6'	0.941 (3)	0.8522 (4)	0.5065 (17)	0.047 (3)	0.490 (16)
H6'	1.1100	0.8403	0.5497	0.057*	0.490 (16)
C7	0.7682 (15)	0.99282 (16)	0.4771 (9)	0.0655 (13)	
H7	0.9180	0.9826	0.5429	0.079*	
C8	0.7245 (14)	1.04208 (16)	0.4601 (8)	0.0570 (14)	
C9	0.5144 (14)	1.0594 (2)	0.3606 (8)	0.0771 (19)	
C10	0.488 (2)	1.1068 (2)	0.3501 (10)	0.107 (3)	
H10	0.3437	1.1187	0.2843	0.129*	
C11	0.6584 (18)	1.1363 (2)	0.4287 (9)	0.0768 (18)	
H11	0.6321	1.1679	0.4172	0.092*	
C12	0.863 (2)	1.1202 (2)	0.5224 (11)	0.102 (3)	
H12	0.9840	1.1404	0.5776	0.122*	
C13	0.8992 (19)	1.0729 (2)	0.5393 (10)	0.111 (3)	
H13	1.0455	1.0618	0.6059	0.133*	

### Atomic displacement parameters (Å^2^)

**Table:**

	*U*^11^	*U*^22^	*U*^33^	*U*^12^	*U*^13^	*U*^23^
S1	0.0310 (6)	0.0410 (5)	0.0369 (5)	0.0050 (6)	−0.0055 (4)	−0.0024 (6)
N1	0.052 (3)	0.041 (2)	0.054 (3)	−0.0006 (18)	0.008 (2)	0.0014 (18)
O1	0.048 (2)	0.0473 (18)	0.0402 (19)	0.0021 (15)	−0.0139 (15)	−0.0081 (15)
O2	0.055 (2)	0.072 (2)	0.070 (3)	0.0037 (18)	0.0028 (19)	0.0086 (19)
O3	0.0328 (18)	0.057 (2)	0.060 (2)	0.0086 (14)	0.0027 (15)	−0.0050 (16)
O4	0.076 (6)	0.062 (3)	0.062 (5)	0.009 (3)	−0.001 (3)	0.008 (4)
O4'	0.076 (6)	0.062 (3)	0.062 (5)	0.009 (3)	−0.001 (3)	0.008 (4)
C1	0.032 (3)	0.040 (2)	0.037 (3)	0.002 (2)	0.003 (2)	0.000 (2)
C2	0.047 (8)	0.039 (6)	0.039 (7)	−0.008 (5)	−0.001 (6)	−0.005 (5)
C3	0.047 (7)	0.045 (6)	0.043 (7)	0.002 (5)	0.001 (6)	0.002 (5)
C2'	0.036 (7)	0.041 (6)	0.047 (8)	−0.004 (5)	−0.004 (6)	−0.004 (5)
C3'	0.044 (7)	0.042 (6)	0.046 (7)	0.005 (5)	0.004 (6)	0.000 (5)
C4	0.041 (3)	0.040 (3)	0.046 (3)	−0.002 (2)	0.005 (2)	−0.001 (2)
C5	0.057 (9)	0.041 (6)	0.042 (7)	0.002 (5)	0.007 (6)	−0.008 (5)
C6	0.048 (8)	0.042 (6)	0.036 (7)	0.002 (5)	−0.003 (6)	−0.007 (5)
C5'	0.041 (8)	0.042 (6)	0.056 (9)	−0.003 (5)	0.004 (7)	−0.008 (6)
C6'	0.037 (8)	0.046 (7)	0.057 (9)	0.003 (5)	0.000 (7)	−0.003 (6)
C7	0.069 (3)	0.048 (3)	0.075 (3)	−0.001 (4)	−0.010 (3)	0.009 (4)
C8	0.064 (4)	0.042 (2)	0.066 (4)	−0.006 (3)	0.013 (3)	0.002 (3)
C9	0.083 (5)	0.051 (3)	0.091 (5)	0.019 (3)	−0.014 (4)	−0.018 (3)
C10	0.125 (7)	0.058 (4)	0.126 (7)	0.030 (4)	−0.035 (6)	−0.003 (4)
C11	0.105 (6)	0.046 (3)	0.084 (5)	0.006 (4)	0.027 (4)	−0.004 (3)
C12	0.118 (7)	0.056 (4)	0.125 (7)	−0.021 (5)	−0.012 (6)	0.001 (5)
C13	0.120 (6)	0.058 (4)	0.139 (7)	−0.013 (4)	−0.042 (6)	0.010 (5)

### Geometric parameters (Å, °)

**Table:**

S1—O1	1.422 (3)	C3'—H3'	0.93
S1—O3	1.428 (3)	C4—C5'	1.359 (15)
S1—O2	1.615 (4)	C4—C5	1.415 (14)
S1—C1	1.767 (4)	C5—C6	1.392 (15)
N1—C7	1.262 (7)	C5—H5	0.93
N1—C4	1.408 (6)	C6—H6	0.93
O2—H2	0.82	C5'—C6'	1.382 (15)
O4—C9	1.359 (10)	C5'—H5'	0.93
O4—H4	0.82	C6'—H6'	0.93
O4'—C9	1.465 (12)	C7—C8	1.451 (6)
O4'—H4'	0.82	C7—H7	0.93
C1—C6	1.340 (11)	C8—C9	1.370 (8)
C1—C2	1.363 (12)	C8—C13	1.372 (9)
C1—C2'	1.405 (12)	C9—C10	1.382 (8)
C1—C6'	1.430 (13)	C10—C11	1.333 (11)
C2—C3	1.348 (13)	C10—H10	0.93
C2—H2A	0.93	C11—C12	1.310 (10)
C3—C4	1.448 (13)	C11—H11	0.93
C3—H3	0.93	C12—C13	1.390 (9)
C2'—C3'	1.387 (14)	C12—H12	0.93
C2'—H2'	0.93	C13—H13	0.93
C3'—C4	1.364 (12)		
			
O1—S1—O3	117.6 (2)	C5—C4—C3	114.6 (7)
O1—S1—O2	108.0 (2)	C6—C5—C4	122.0 (10)
O3—S1—O2	106.9 (2)	C6—C5—H5	119.0
O1—S1—C1	108.29 (19)	C4—C5—H5	119.0
O3—S1—C1	106.8 (2)	C1—C6—C5	117.5 (10)
O2—S1—C1	108.9 (2)	C1—C6—H6	121.2
C7—N1—C4	121.3 (4)	C5—C6—H6	121.2
S1—O2—H2	109.5	C4—C5'—C6'	118.2 (11)
C9—O4—H4	109.5	C4—C5'—H5'	120.9
C9—O4'—H4'	109.5	C6'—C5'—H5'	120.9
C6—C1—C2	125.4 (8)	C5'—C6'—C1	121.1 (11)
C6—C1—C2'	109.3 (8)	C5'—C6'—H6'	119.5
C2—C1—C6'	108.3 (7)	C1—C6'—H6'	119.5
C2'—C1—C6'	116.8 (7)	N1—C7—C8	123.2 (6)
C6—C1—S1	116.8 (6)	N1—C7—H7	118.4
C2—C1—S1	117.7 (5)	C8—C7—H7	118.4
C2'—C1—S1	121.8 (5)	C9—C8—C13	117.8 (6)
C6'—C1—S1	121.4 (5)	C9—C8—C7	121.3 (6)
C3—C2—C1	117.3 (10)	C13—C8—C7	120.9 (6)
C3—C2—H2A	121.4	O4—C9—C8	121.9 (6)
C1—C2—H2A	121.4	O4—C9—C10	117.7 (7)
C2—C3—C4	123.0 (10)	C8—C9—C10	117.7 (6)
C2—C3—H3	118.5	C8—C9—O4'	116.2 (6)
C4—C3—H3	118.5	C10—C9—O4'	122.7 (7)
C3'—C2'—C1	121.9 (9)	C11—C10—C9	123.8 (7)
C3'—C2'—H2'	119.0	C11—C10—H10	118.1
C1—C2'—H2'	119.0	C9—C10—H10	118.1
C4—C3'—C2'	117.6 (10)	C12—C11—C10	119.1 (7)
C4—C3'—H3'	121.2	C12—C11—H11	120.5
C2'—C3'—H3'	121.2	C10—C11—H11	120.5
C5'—C4—C3'	124.4 (8)	C11—C12—C13	119.9 (8)
C5'—C4—N1	121.3 (6)	C11—C12—H12	120.0
C3'—C4—N1	114.1 (6)	C13—C12—H12	120.0
C3'—C4—C5	109.2 (8)	C8—C13—C12	121.7 (8)
N1—C4—C5	126.3 (6)	C8—C13—H13	119.2
C5'—C4—C3	106.8 (8)	C12—C13—H13	119.2
N1—C4—C3	119.1 (6)		
			
O1—S1—C1—C6	173.5 (9)	C3'—C4—C5—C6	34.7 (15)
O3—S1—C1—C6	−58.9 (9)	N1—C4—C5—C6	177.2 (9)
O2—S1—C1—C6	56.3 (9)	C3—C4—C5—C6	−3.6 (16)
O1—S1—C1—C2	−7.4 (9)	C2—C1—C6—C5	−0.2 (17)
O3—S1—C1—C2	120.2 (8)	C2'—C1—C6—C5	−37.8 (14)
O2—S1—C1—C2	−124.6 (8)	C6'—C1—C6—C5	71.4 (15)
O1—S1—C1—C2'	35.0 (9)	S1—C1—C6—C5	178.8 (9)
O3—S1—C1—C2'	162.6 (8)	C4—C5—C6—C1	3.0 (18)
O2—S1—C1—C2'	−82.2 (9)	C3'—C4—C5'—C6'	−2.4 (19)
O1—S1—C1—C6'	−144.8 (9)	N1—C4—C5'—C6'	−178.4 (10)
O3—S1—C1—C6'	−17.2 (9)	C5—C4—C5'—C6'	71.8 (15)
O2—S1—C1—C6'	98.0 (9)	C3—C4—C5'—C6'	−37.3 (15)
C6—C1—C2—C3	−1.7 (17)	C4—C5'—C6'—C1	1(2)
C2'—C1—C2—C3	72.3 (14)	C6—C1—C6'—C5'	−86.4 (17)
C6'—C1—C2—C3	−38.2 (14)	C2—C1—C6'—C5'	39.1 (16)
S1—C1—C2—C3	179.3 (9)	C2'—C1—C6'—C5'	0.2 (17)
C1—C2—C3—C4	1.0 (18)	S1—C1—C6'—C5'	180.0 (10)
C6—C1—C2'—C3'	38.5 (15)	C4—N1—C7—C8	−178.4 (6)
C2—C1—C2'—C3'	−85.4 (15)	N1—C7—C8—C9	−2.7 (11)
C6'—C1—C2'—C3'	−0.5 (16)	N1—C7—C8—C13	179.9 (8)
S1—C1—C2'—C3'	179.7 (9)	C13—C8—C9—O4	159.1 (9)
C1—C2'—C3'—C4	−0.6 (17)	C7—C8—C9—O4	−18.5 (11)
C2'—C3'—C4—C5'	2.1 (17)	C13—C8—C9—C10	−1.8 (11)
C2'—C3'—C4—N1	178.4 (9)	C7—C8—C9—C10	−179.4 (8)
C2'—C3'—C4—C5	−34.2 (14)	C13—C8—C9—O4'	−161.6 (8)
C2'—C3'—C4—C3	71.5 (13)	C7—C8—C9—O4'	20.9 (11)
C7—N1—C4—C5'	−19.9 (12)	O4—C9—C10—C11	−160.3 (10)
C7—N1—C4—C3'	163.7 (9)	C8—C9—C10—C11	1.4 (14)
C7—N1—C4—C5	22.8 (12)	O4'—C9—C10—C11	159.7 (10)
C7—N1—C4—C3	−156.4 (9)	C9—C10—C11—C12	−0.2 (15)
C2—C3—C4—C5'	38.7 (16)	C10—C11—C12—C13	−0.4 (14)
C2—C3—C4—C3'	−87.5 (16)	C9—C8—C13—C12	1.3 (13)
C2—C3—C4—N1	−179.1 (10)	C7—C8—C13—C12	178.9 (9)
C2—C3—C4—C5	1.5 (17)	C11—C12—C13—C8	−0.2 (14)
C5'—C4—C5—C6	−88.1 (15)		

### Hydrogen-bond geometry (Å, °)

**Table:**

*D*—H···*A*	*D*—H	H···*A*	*D*···*A*	*D*—H···*A*
O2—H2···O3^i^	0.82	2.17	2.917 (5)	151
O4—H4···N1	0.82	2.01	2.665 (10)	136

Symmetry codes: (i) *x*−1, *y*, *z*.
